# Dubin-Johnson Syndrome Presenting During Cardiac Transplantation Evaluation

**DOI:** 10.7759/cureus.6594

**Published:** 2020-01-08

**Authors:** Alexis LeVee, Craig Cooper, Michael B Russell, Mark Sterling

**Affiliations:** 1 Internal Medicine, Cedars-Sinai Medical Center, Los Angeles, USA; 2 Internal Medicine, Tufts Medical Center, Boston, USA; 3 Internal Medicine: Gastroenterology, Tufts Medical Center, Boston, USA

**Keywords:** dubin johnson syndrome, hyperbilirubinemia, cardiac surgery, liver biopsy

## Abstract

Dubin-Johnson syndrome is a rare, benign disorder that results in conjugated hyperbilirubinemia. The disease manifests as intermittent jaundice without long-term hepatic or other clinical complications. This article reports a case of Dubin-Johnson syndrome, which was identified during cardiac transplant evaluation for cardiomyopathy secondary to a polyglycogen storage disease. The patient successfully underwent an orthotopic heart transplant. Postoperatively, her conjugated hyperbilirubinemia increased as compared to her baseline but resolved after several weeks. This report briefly reviews the hepatic manifestations in patients with Dubin-Johnson syndrome undergoing major surgery and highlights urinary coproporphyrin as a useful diagnostic test for Dubin-Johnson syndrome.

## Introduction

Dubin-Johnson syndrome is a rare condition that results from a mutation in the transporter protein that excretes conjugated bilirubin out of hepatocytes [1]. The defect causes conjugated hyperbilirubinemia and grossly black livers without evidence of long-term clinical consequences. The features of the syndrome include an elevated conjugated hyperbilirubinemia in the absence of other hepatic abnormalities, an increased ratio of urinary coproporphyrin I to coproporphyrin III, and a liver biopsy showing a centrilobular deposition of dark pigment [2]. This article describes a patient with polyglycogen storage cardiomyopathy requiring a cardiac transplant who was found to have Dubin-Johnson syndrome during the preoperative workup of elevated liver function tests. The hepatic manifestations of Dubin-Johnson syndrome in patients undergoing major surgery, as well as the utility of urinary coproporphyrin testing for the workup of unexplained direct hyperbilirubinemia, is briefly reviewed.

## Case presentation

A 25-year-old female with a medical history of infiltrative non-ischemic cardiomyopathy secondary to a polyglycogen storage disease on the United Network for Organ Sharing (UNOS) 1B heart transplant list presented with four days of fever, nausea, vomiting, and diarrhea. She denied abdominal pain or pruritus. The patient is of Palestinian descent with no history of liver disease in her family. She reported no alcohol or drug use and there was no history of a prior blood transfusion. She had one episode of jaundice during a viral illness in the last year. On examination, the patient was afebrile with a blood pressure of 89/54 and a heart rate of 98. She appeared jaundiced with scleral icterus. The abdominal exam was soft and non-tender with no hepatosplenomegaly.

Laboratory values were significant for an elevated leukocyte count of 11.6 K/uL and low hemoglobin and hematocrit of 9.6 g/dL and 27.5%, respectively. Her liver function tests (LFTs) performed on admission were significant for aspartate aminotransferase (AST) of 6114 IU/L and alanine aminotransferase (ALT) of 3606 IU/L, which were presumed to be elevated due to volume loss with underlying cardiac dysfunction, and a total bilirubin of 27.5 mg/dL and direct bilirubin of 19.3 mg/dL. The patient was ultimately found to have positive blood cultures for Serratia marcescens, believed to be due to a peripherally inserted central catheter (PICC) line infection. After treatment with antibiotics and hydration, her serum aminotransferase levels returned to normal levels while her total bilirubin and direct bilirubin remained elevated in the range of 3-6 mg/dL and 3-5 mg/dL, respectively. Her viral markers for hepatitis and serologies for autoimmune hepatitis were negative. An abdominal ultrasound and a CT scan of the abdomen revealed no significant findings in the liver or gallbladder. Prior to this admission, a few liver function tests (LFTs) were obtained, but her baseline aminotransferase levels appeared to be normal while her baseline total bilirubin and direct bilirubin were approximately 3.5 mg/dL and 2.0 mg/dL, respectively.

**Figure 1:**
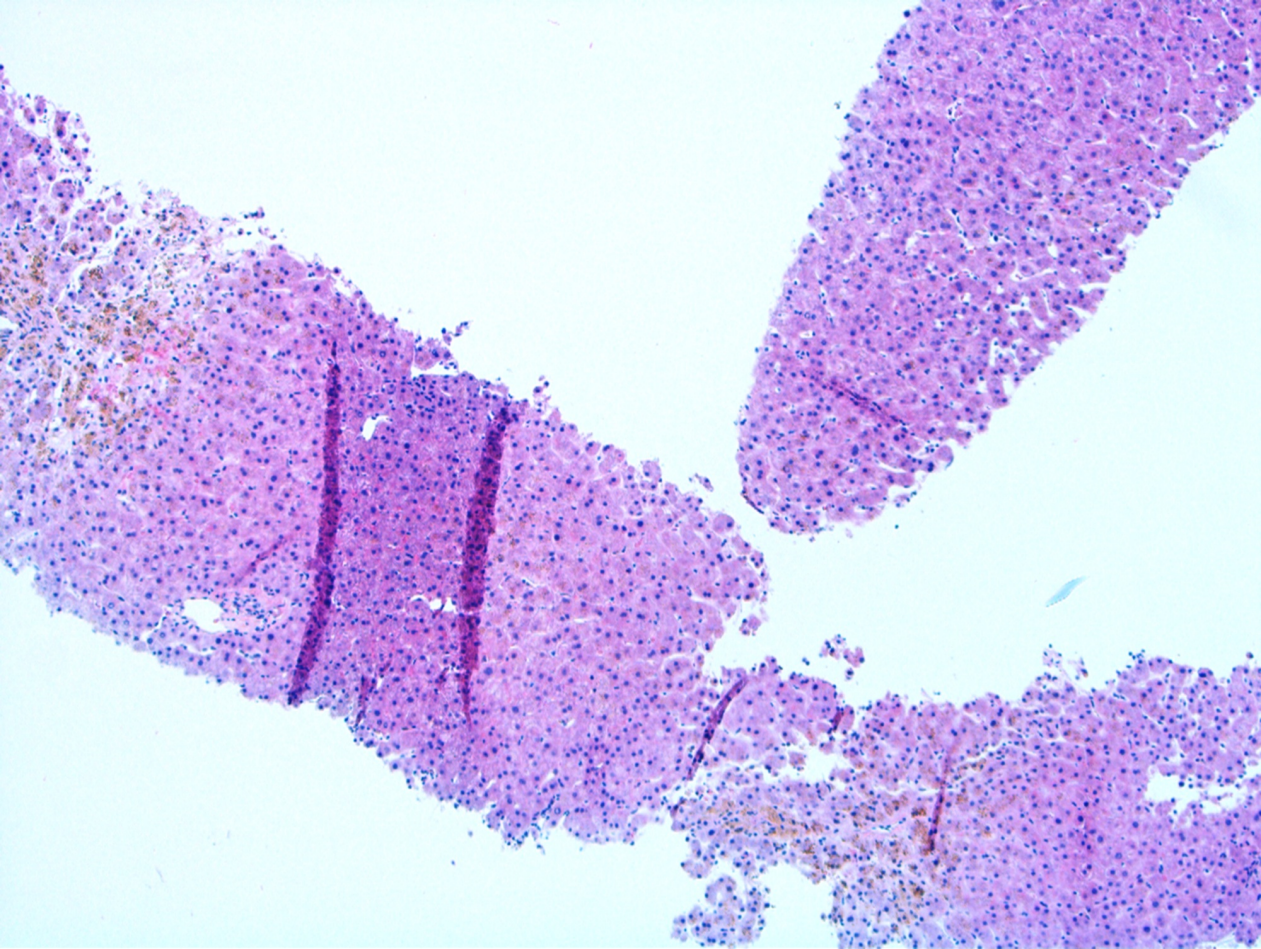
Liver histology showed mild centrilobular fibrosis with significant intracellular pigment in the centrilobular hepatocytes Hematoxylin and eosin stain x 10

**Figure 2:**
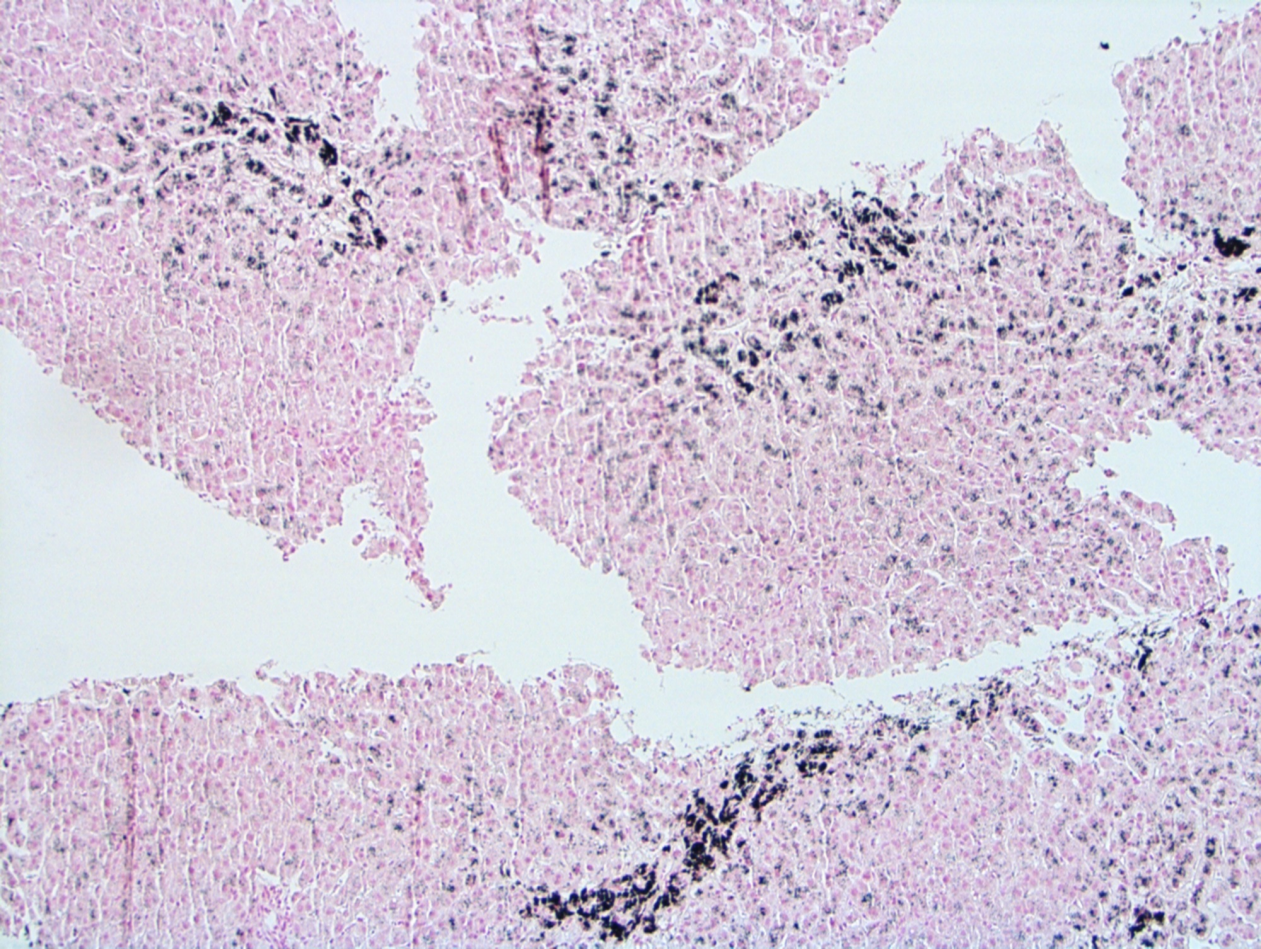
Intracellular pigment is black on Fontana-Masson stain Fontana-Masson stain x 10

Given her elevated LFTs of unclear origin, she underwent a liver biopsy as part of her cardiac transplant evaluation, which showed mild centrilobular fibrosis with marked centrilobular intrahepatocellular pigment present (Figure 1). The pigment was positive on Periodic acid-Schiff-diastase (PAS-D) and Fontana stains (Figure 2) and negative on acid-fast bacilli (AFB) and iron stains, confirming the diagnosis of Dubin-Johnson syndrome.

The patient’s hospital course was complicated by decompensated heart failure, requiring increasing doses of inotropic support. She successfully underwent orthotopic heart transplantation three weeks later. On the day after the surgery, her LFTs revealed AST of 133 IU/L, ALT of 40 IU/L, total bilirubin of 6.5 mg/dL, and direct bilirubin of 4.8 mg/dL. Her hemoglobin was 11.9 g/dL and hematocrit was 34.1%. The total and direct bilirubin peaked the day after the surgery and eventually returned to her baseline after two weeks.

## Discussion

The diagnosis of Dubin-Johnson syndrome was made in our patient based on the characteristic LFTs and biopsy findings of the disease. In Dubin-Johnson syndrome, the serum total bilirubin concentrations are typically elevated, usually ranging from 2 to 5 mg/dl, but is over 10 mg/dl in 5% of the reported cases. Direct bilirubin accounts for about 60% of this elevation [3]. The remaining LFTs are normal. Liver biopsy reveals a coarsely granular brown pigment in the hepatic parenchymal cells, primarily in a centrilobular distribution. The pigment stains positive for the Fontana-Masson and PAS stains and negative for Prussian blue [4].

Dubin-Johnson syndrome can present insidiously but is often precipitated by other diseases or by periods of major stress, including infection, pregnancy, and surgery. Postoperatively, a predominantly conjugated hyperbilirubinemia develops with otherwise normal hepatic function, often leading to transient jaundice [3]. In most cases, serum bilirubin levels return to baseline postoperatively and there are no long-term hepatic or other clinical consequences.

There are several reports of Dubin-Johnson syndrome after open-heart surgery, specifically, which display a similar clinical picture; however, trauma to red blood cells from a cardiopulmonary bypass and intravascular hemolysis secondary to prosthetic valves can cause an even greater increase in postoperative hyperbilirubinemia [5-6]. Our patient’s clinical course was consistent with this, revealing an increased direct hyperbilirubinemia immediately post the operation without other hepatic consequences and returning to baseline after several weeks. In addition, Singh and Baker (1974) showed that preoperative hepatic function is not correlated with postoperative jaundice following open-heart surgery [7]. Accordingly, an elevation in bilirubin levels in Dubin-Johnson syndrome prior to surgery is not a predisposing factor to postoperative jaundice. Our patient did not display jaundice post the operation despite increased bilirubin levels prior to surgery.

This case highlights the importance of considering Dubin-Johnson syndrome in the presence of conjugated hyperbilirubinemia despite other confounding comorbidities. To our knowledge, this is the first reported case of a patient with Dubin-Johnson syndrome and a glycogen storage disease in the literature, and no connection between the diseases is known. Because the patient had a polyglycogen storage myopathy, when LFTs were found to be abnormal, a liver biopsy was pursued. However, the liver biopsy could have potentially been avoided if Dubin-Johnson syndrome was considered previously and a urinary coproporphyrin was obtained.

An abnormal urinary coproporphyrin is diagnostic in patients with Dubin-Johnson syndrome. Coproporphyrin is excreted in two types of isomers in the urine, I and III, and, normally, people excrete about 75% of coproporphyrin isomer III in the urine [2]. However, patients with Dubin-Johnson syndrome excrete 80% of coproporphyrin I in urine with normal total coproporphyrin excretion [2]. Thus, it is important to recognize an isolated conjugated hyperbilirubinemia despite rare comorbidities and to obtain a urinary coproporphyrin test to potentially avoid liver biopsy and any further diagnostic workup. 

## Conclusions

Dubin-Johnson syndrome results in a direct conjugated hyperbilirubinemia with otherwise normal hepatic function. Postoperatively, patients can have markedly increased levels of hyperbilirubinemia, secondary to stress from surgery and intravascular hemolysis if a cardiopulmonary bypass is used, which often returns to baseline without long-term hepatic damage or other clinical consequences. The urinary coproporphyrin test is diagnostic in patients with Dubin-Johnson syndrome and should be considered to avoid any further diagnostic workup.
